# Infant study of hemispheric asymmetry after long‐gap esophageal atresia repair

**DOI:** 10.1002/acn3.51465

**Published:** 2021-10-18

**Authors:** Mackenzie S. Kagan, Chandler R. L. Mongerson, David Zurakowski, Russell W. Jennings, Dusica Bajic

**Affiliations:** ^1^ Department of Anesthesiology Critical Care and Pain Medicine Boston Children’s Hospital 300 Longwood Ave. Boston Massachusetts 02115 USA; ^2^ Harvard Medical School Harvard University 25 Shattuck St. Boston Massachusetts 02115 USA; ^3^ Department of Surgery Boston Children’s Hospital 300 Longwood Ave. Boston Massachusetts 02115 USA; ^4^ Esophageal and Airway Treatment Center Boston Children’s Hospital 300 Longwood Ave. Boston Massachusetts 02115 USA

## Abstract

**Objectives:**

Previous studies have demonstrated that infants are typically born with a *left‐greater‐than‐right* forebrain asymmetry that reverses throughout the first year of life. We hypothesized that critically ill term‐born and premature patients following surgical and critical care for long‐gap esophageal atresia (LGEA) would exhibit alteration in expected forebrain asymmetry.

**Methods:**

Term‐born (*n* = 13) and premature (*n* = 13) patients, and term‐born controls (*n* = 23) <1 year corrected age underwent non‐sedated research MRI following completion of LGEA treatment via Foker process. Structural T1‐ and T2‐weighted images were collected, and ITK‐SNAP was used for forebrain tissue segmentation and volume acquisition. Data were presented as *absolute* (cm^3^) and *normalized* (% total forebrain) volumes of the hemispheres. All measures were checked for normality, and group status was assessed using a general linear model with age at scan as a covariate.

**Results:**

Absolute volumes of both forebrain hemispheres were smaller in term‐born and premature patients in comparison to controls (*p* < 0.001). Normalized hemispheric volume group differences were detected by T1‐weighted analysis, with premature patients demonstrating right‐greater‐than‐left hemisphere volumes in comparison to term‐born patients and controls (*p* < 0.01). While normalized group differences were very subtle (a right hemispheric predominance of roughly 2% of forebrain volume), they represent a deviation from the expected pattern of hemispheric brain asymmetry.

**Interpretation:**

Our pilot quantitative MRI study of hemispheric volumes suggests that premature patients might be at risk of altered expected *left‐greater‐than‐right* forebrain asymmetry following repair of LGEA. Future neurobehavioral studies in infants born with LGEA are needed to elucidate the functional significance of presented anatomical findings.

## Introduction

Previous morphometric studies have demonstrated that otherwise healthy infants are born with a *left‐greater‐than‐right* forebrain hemispheric asymmetry that reverses throughout the first year of life to match the characteristic\x92right‐greater‐than‐left asymmetry observed in adults.^1^, ^2^, ^3^, ^4^ This asymmetry was reported in large‐scale volumetric structures such as the cerebral hemispheres, lateral ventricles, and subcortical gray matter volumes.^1^ Diffusion tensor imaging has also shown leftward asymmetries in white matter bundles in language and motor‐related fibers and revealed greater structural efficiency in the left hemisphere.^1^, ^2^ The magnitude of such asymmetry is larger in neonates than adults, suggesting that the characteristic reversed asymmetry seen in adults is not present at birth, but rather is developed over time.^1^, ^3^, ^5^, ^6^ The switch in asymmetry around one year of life is attributed to rapid growth of cortical gray matter in the right hemisphere, likely due to the vast increase in new cortical synapses^1^ resulting in a larger right hemisphere later in life.

Alterations in *left‐greater‐than‐right* hemispheric asymmetry of infancy have been reported in a variety of neurodevelopmental disorders.^7^, ^8^, ^9^ However, there is a gap in our understanding of whether critical illness in infancy may alter this asymmetry that has been otherwise linked with normal lateralization of motor and cognitive functions.^10^, ^11^ Stressors such as procedural pain and prolonged exposure to analgesic medications may result in altered brain microstructure in premature infants at term‐equivalent age when compared to normal brain growth that is independent of the degree of prematurity.^12^ Emerging reports also suggest that infants born with noncardiac congenital anomalies undergoing surgery and complex critical care in infancy are at increased risk of brain injury^13^, ^14^ and poor long‐term outcomes.^15^, ^16^ However, these studies did not assess hemispheric asymmetry. Considering the most dynamic brain growth occurs in the first year of life^1^ exposure to critical illness may pose a risk to alterations in expected *left‐greater‐than‐right* hemispheric asymmetry of infancy.

Our recent pilot study reported clinically significant incidental MRI brain findings, as well as globally smaller brain size^13^, ^17^ and potentially delayed brain growth^18^ in a pilot cohort of term‐born and premature infants born with long‐gap esophageal atresia (LGEA) following complex perioperative care. In the same pilot group, we also showed disproportionally smaller corpus callosum,^19^ implicating structural (mal)adaptations of the forebrain not evident with gross forebrain analysis.^17^ The unique aspect of selected cohort is that these critically ill infants born with LGEA underwent complex perioperative critical care involving tension‐induced esophageal growth known as the Foker process,^20^, ^21^, ^22^ requiring prolonged sedation ≥5 days leading to physical dependence on the drugs of sedation.^23^, ^24^ We hypothesized that when compared to healthy infants, both critically ill term‐born and premature patients following critical care for LGEA with Foker process would exhibit alteration in the expected *left‐larger‐than‐right* hemispheric asymmetry of infancy. Therefore, this report addresses the possible (mal)adaptation in hemispheric asymmetry in the same, aforementioned cohort using structural T1‐^19^ and T2‐weighted^17^ brain magnetic resonance imaging (MRI).

## Methods

### Study design and participants

This pilot MRI study builds on our previous reports^13^, ^17^, ^19^ using data from the same infant study cohort. Our study received ethical approval from Boston Children’s Hospital Institutional Review Board as a “no more than minimal risk” study and recruitment was possible thanks to *The Esophageal and Airway Treatment Center* at Boston Children’s Hospital ‐ a premier program designed to treat infants born with thoracic noncardiac and gastrointestinal congenital anomalies, especially LGEA. A representative timeline illustrating the sequence of perioperative critical care for Foker process^20^ was previously presented,^18^, ^23^ while associations between individual MRI end‐point measures (e.g., number of cranial MRI findings and brain volumes) and the clinical measures of care as to assess the severity of underlying disease in cohort subjects will be presented elsewhere. Methodological approach for recruitment criteria and MRI scanning were previously described.^13^, ^17^, ^19^ Briefly, informed written parental consent was obtained for non‐sedated research brain MRI participation, in accordance with the Declaration of Helsinki and Good Clinical Practice guidelines. The family of each subject received a $90 gift card following the completion of the scan.

Eligibility criteria included both term‐born (37–42 weeks gestational age (GA) at birth) and moderate‐to‐late preterm (28–36 weeks GA at birth) patients <1 year gestation‐corrected age that underwent Foker process for LGEA repair (n = 13/patient group). Exclusion criteria included: (1) extreme prematurity (<28 weeks GA); (2) extracorporeal membrane oxygenation (ECMO) exposure; (3) clinically indicated cranial ultrasound findings (e.g. ventricular enlargement with or without gray matter and/or ventricular hemorrhage); (4) neurological disease (e.g., seizures) as documented in clinical records; (5) chromosomal abnormalities (e.g., Down syndrome); (6) prenatal drug exposure to either drugs of abuse or prescription medications; and/or (7) MRI incompatible implants. Indeed, we recruited only those patients born with LGEA that had no clinical evidence of neurogical problems at the time of recruitment as per detailed chart review. Healthy term‐born infants <1 year old with no prior exposure to surgery, anesthesia, or sedation were recruited from a pool of Boston Children’s Hospital outpatients and two neighboring newborn centers (Beth Israel Deaconess Medical Center and Brigham and Women’s Hospital) and served as a reference baseline for typical forebrain and hemispheric volumes that were not age or gender matched. Updated and comprehensive summary of recruitment details and final group characteristics is described below and summarized in Table 1.

**Table 1:** Recruitment and group characteristics.

1. Recruitment Process	Term‐born controls		Term‐born patients	Preterm patients
Considered/(Chart) Reviewed	63		173	108
Eligible (%Reviewed)	60 (95%)		63 (36%)	49 (45%)
Approached (%Eligible)	57 (95%)		40 (63%)	23 (47%)
Consented (%Approached)	23 (40%)		19 (48%)	18 (78%)
Scanned (%Consented)	23 (100%)		13 (68%)	13 (72%)
**Included/Analyzed (%Scanned** **)**	**22 (96%)**		**13 (100%)**	**13 (100%)**
**2. Group Characteristics by MRI Analysis**	**T1‐weighted** **(*n* = 20)**	**T2‐weighted** **(*n* = 17)**	**Both scans** **(*n* = 13)**	**Both scans** **(*n* = 13)**
Sex (male), n (%)	16 (80%)	14 (82%)	7 (54%)	8 (62%)
GA at birth (weeks), Mean ± SD	39.3 ± 1.1	39.3 ± 1.1	38.5 ± 1.1	32.2 ± 2.9
CA at scan (months), Median [range]	4.5 [0.5‐12.3]	3.2 [0.5‐9.3]	5.4 [0.7‐13.0]	3.8 [1.4‐7.5]
Twin births, n (%)	1 (5%)	1 (6%)	1 (8%)	2 (15%)
Primary diagnoses				
Isolated LGEA, n (%)	0	0	3 (23%)	3 (23%)
LGEA with TEF, n (%)	0	0	5 (38%)	9 (69%)
Other, n (%)	0	0	5 (38%)	1 (8%)

Table summarizes **(1)** study recruitment process for the 3 groups (term‐born healthy controls, and term‐born and preterm patients), as well as **(2)** group characteristics of all subjects included in the quantitative T1‐ and T2‐weighted structural analyses. Bold values indicate that MRI data from 100% of term‐born and premature patients, and 96% of controls were included in the analysis, a testament of successful non‐sedated research brain MRI. Numbers for recruitment process are updated since our previous reports for T1‐,^19^ and T2‐weighted^17^ analysis (see Methods). Primary diagnoses included: (1) isolated long‐gap esophageal atresia (LGEA), (2) LGEA with tracheo‐esophageal fistula (TEF), or (3) other that included TEF as part of VACTERL association (without cardiac component). Infants diagnosed with VACTERL typically exhibit ≥3 of the characteristic features (viz. **V**ertebral defects; **A**nal atresia; **C**ardiac defects; **T**racheo‐**E**sophageal fistula; **R**enal anomalies; **L**imb abnormalities). None of the infants included in analysis were exposed to extracorporeal membrane oxygenation. For other exclusion criteria, see Methods.

Abbreviations: CA, corrected age; GA, gestational age.

### MRI acquisition

Our MRI scanning protocol was previously described in detail.^13^, ^17^, ^19^ Briefly, all infants underwent a *non‐sedated* research brain MRI scan after completion of all post‐operative critical care for Foker process using a ‘feed and wrap’ approach.^25^, ^26^, ^27^, ^28^ Corrected age at scan for all cohort subjects was calculated as follows: postnatal age (weeks) – [40 – gestational age at birth (weeks)]. Patients were scanned in late evenings or at night using a 3T TrioTim MRI system equipped with 32‐channel receive‐only head coil and body‐transmission (Siemens Healthcare Inc., USA). All infants were continuously monitored for stable heart rate and oxygenation throughout MRI acquisition. Total number of scans included in the analysis per group is summarized in Table 1.

#### Structural T1‐weighted MRI

**Figure 1:**
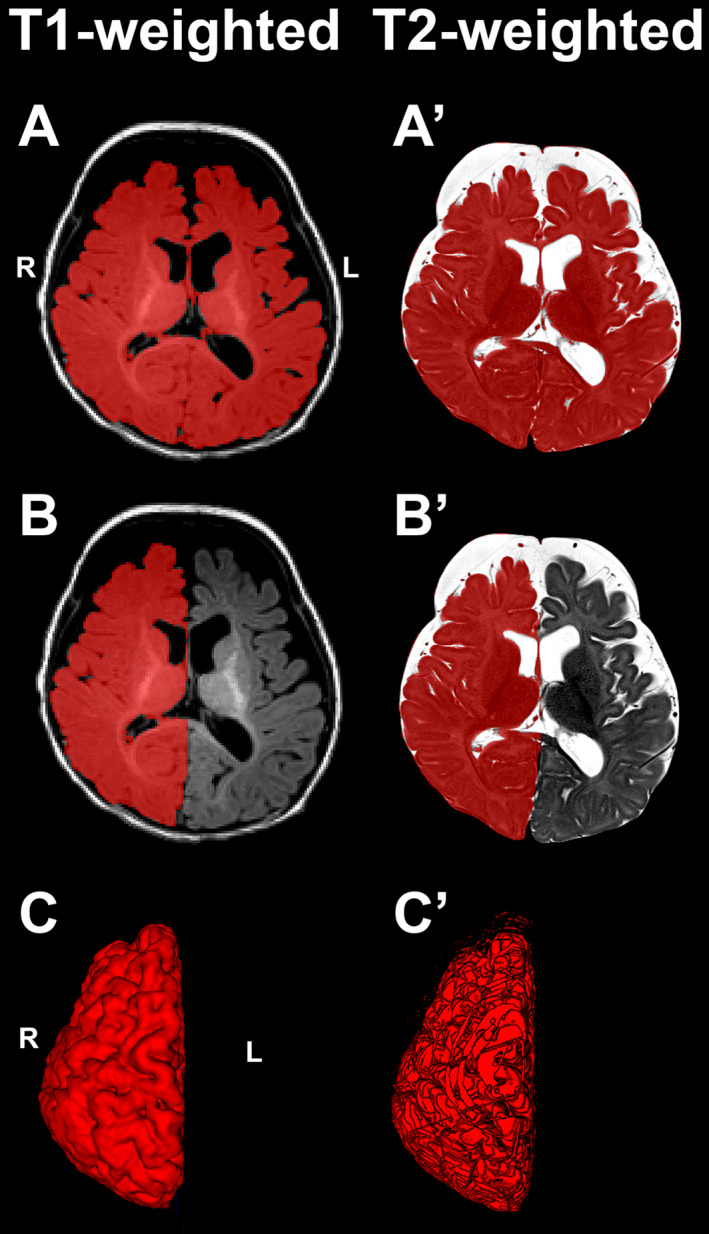
Total and Hemispheric Forebrain Segmentation. Representative segmentation masks of the forebrain and right hemisphere are shown in axial sections of T1‐ (Panel A and B) and T2‐weighted images (Panel A’ and B’), respectively. Panels B and B’ show the left hemisphere segmentation erased from the total forebrain mask. Bottom panels illustrate 3D renderings of right hemispheres based on T1‐ (C) and T2‐weighted (C’) MRI segmentation. Note the slight difference in 3D space resulting from the two different structural MRI modalities. For more details on differences between two MRI modalities, refer to Table 2.

Images were acquired using a MPRAGE sequence (repetition time = 2.52 s; echo time = 1.74 ms; flip angle = 7°; field of view = 192 × 192 mm^2^; voxel size = 1 × 1 × 1 mm^3^; 144 sagittal slices). T1‐weighted images (Fig. 1A and B) were collected for all scanned term‐born and preterm patients (n = 13/group), and 21/23 (91%) term‐born controls (Table 1). Since our recent report,^19^ we added one additional T1‐weighted control scan. Of those 21 controls, only one infant had partial brain coverage that precluded analysis of forebrain volume (*n* = 20 controls for T1‐weighted analysis). We noted minor ringing artifact due to motion only in 1/20 controls and 1/13 premature patients that did not obscure forebrain delineation and segmentation.

#### Structural T2‐weighted MRI

Images were acquired using an axial fast spin‐echo sequence (repetition time = 12.62 s; echo time = 110 ms; flip angle = 120°; field of view = 180 × 180 mm^2^; voxel size = 0.35 × 0.35 mm^2^; 63 slices of 2 mm thickness). T2‐weighted images (Fig. 1A’ and B’) were collected for all scanned term‐born and preterm patients (*n* = 13/group), and 18/23 (78%) term‐born controls. T2‐weighted analysis is an extension of our previous work^17^ with the addition of two new control subjects (4‐ and 5‐month‐old). Additionally, replacement follow‐up scans for two previously analyzed infants^17^ were substituted to improve the quality of T2‐weighted images (3 month‐old control subject and 5 month‐old term‐born patient). Of the 18 controls, only one infant had partial brain coverage that precluded analysis of forebrain volumes (*n* = 17 controls for T2‐weighted analysis). We noted very minor ringing artifact or single slice disruption due to head motion in a limited number of infants: 2/17 controls, 2/13 term‐born patients, and 3/13 preterm patients. Given incidences were roughly evenly dispersed across groups and the low probability that such minor artifacts would impact gross brain volume estimations, the decision was made to include these subjects. In the case of an artifact‐corrupted slice, segmentations were easily approximated using 3D rendering tool in ITK‐SNAP software (v.3.6.0; www.itksnap.org).^29^

### Quantitative MRI analyses

**Figure 2:**
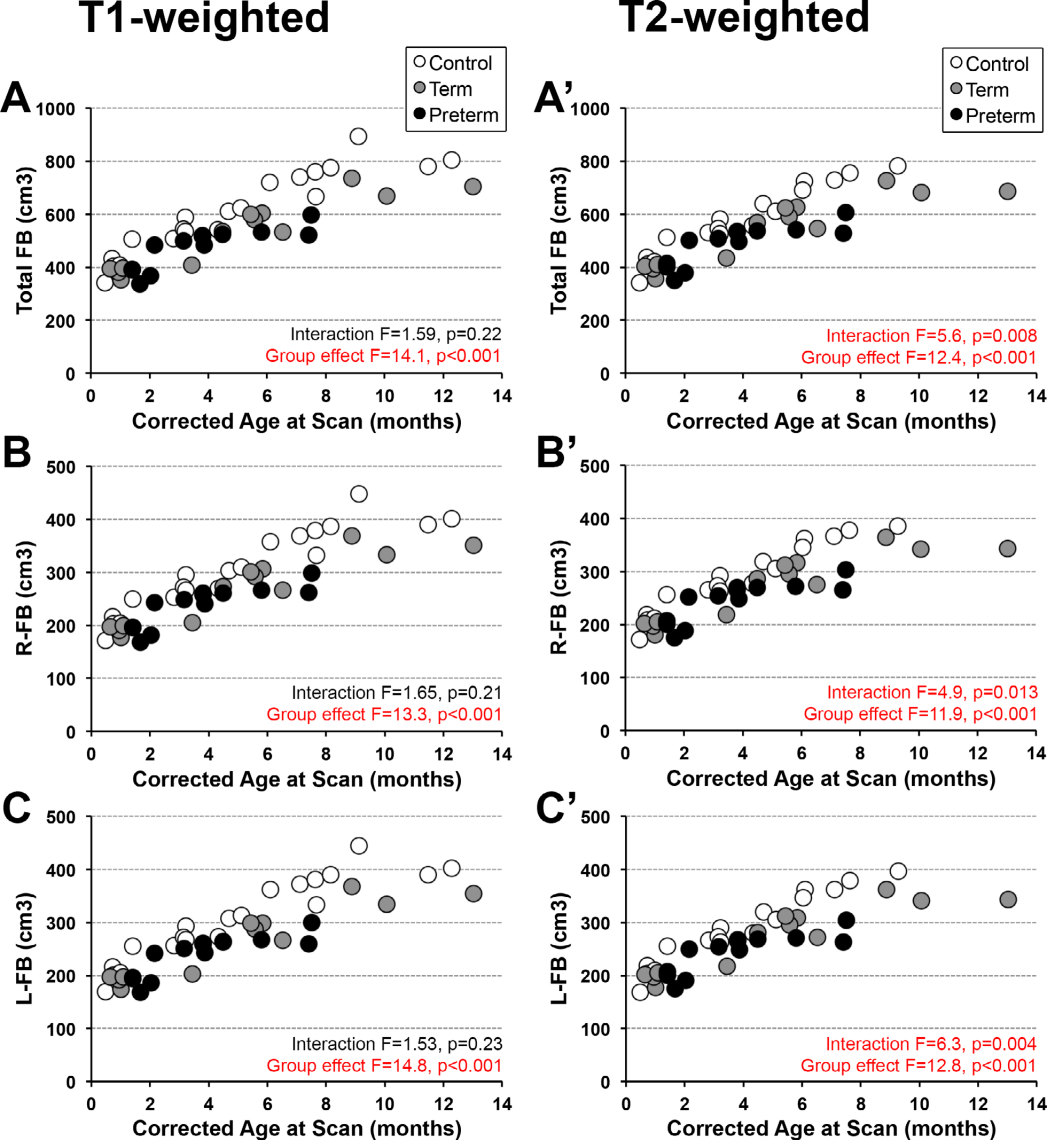
Absolute Forebrain and Hemispheric Volumes. Graphs show individual absolute volumes (cm^3^) of the total forebrain (FB; A and A’), as well as left (L; B and B’) and right (R; C and C’) hemispheres using T1‐ (A‐C) and T‐2 weighted (A’‐C’) data analysis for the 3 groups: (1) term‐born controls (open circles), (2) term‐born patients (gray circles), and (3) premature patients (black circles). Note a significant increase in absolute volumes for all groups with age (see Results section for statistical details). No significant interactions between age at scan and group status were found for T1‐weighted data analysis (A‐C), which was in contrast to the significant interaction observed for T2‐weighted data analysis (A’‐C’) suggesting altered growth trajectories between groups. Uniformly, absolute values for forebrain (A and A’), right hemisphere (B and B’) and left hemisphere (C and C’) volumes were significantly smaller in both term‐born and premature patients in comparison to controls (both *p* < 0.001), with no difference between patient groups (*p* > 0.7). *Abbreviations:* FB, forebrain; L, left; R, right.

To strengthen confidence in our findings, hemispheric volume analysis was performed using both T1‐ and T2‐weighted MRI data. T1‐weighted images offered better overall resolution (1 mm^3^ isotropic voxels), and more T1‐weighted scans were available for controls (including those at older ages), whereas the T2‐weighted images provided superior in‐plane resolution (0.35 × 0.35 × 2.0 mm^3^ voxels) and tissue contrasts. Table 2 summarizes the difference between two different structural MRI modalities. To correct for any head tilt in structural images, brains were aligned along the anterior commissure ‐ posterior commissure (AC‐PC) line using Freeview (v.2.0; *see Fig. 2 in* Ref. [13]) prior to subsequent total brain segmentation.

**Table 2:** T1‐ and T2‐weighted MRI analysis differences.

	T1‐weighted Analysis	T2‐weighted Analysis
*Tissue Contrasts Between Grey and White Matter*	Poorer* tissue contrast in infants^46^ (*Not impactful for gross forebrain analysis)	Better tissue contrast in infants^46^
*Voxel Size*	1 × 1 × 1 mm^3^ Better overall resolution for gross 3D estimation (see Fig. 1C)	0.35 × 0.35 × 2.0 mm^3^ Better in‐plane resolution, but poorer overall resolution for gross 3D estimation (see Fig. 1C’)
*Segmentation: Elimination of CSF Methodology*	FMRIB’s Automated Segmentation Tool (FAST) required subsequent manual editing of the whole brain, CSF, cerebellum, and brainstem (Greater possibility of individual bias)	1. Morphologically Adaptive Neonatal Tissue Segmentation (MANTiS) created automatic brain tissue masks that required only additional minor manual editing (Less possibility of individual bias) 2. Clearer CSF to GM/WM differentiation (Negligible impact due to greater overall resolution of T1‐weighted images)
*Volume Extraction*	ITK‐SNAP software	

Table summarizes the main methodological considerations when evaluating results of T1‐ and T2‐weighted MRI analyses in this study. For further details on differences in methodology for infant brain segmentation, see *Methods* section.

Abbreviations: CSF, cerebrospinal fluid; GM, grey matter; WM, white matter.

#### T1‐weighted total brain segmentation

As previously described,^19^ we performed semi‐automated *total brain* tissue segmentation of T1‐weighted MRI images which included several preprocessing steps: (i) Skull‐stripping of T1 images by manually tracing whole‐brain outline (includes ventricular system); and (ii) Partial volume segmentation of cerebrospinal fluid (CSF) using **F**MRIB’s **A**utomated **S**egmentation **T**ool (FAST).^30^ Using tools in **F**MRIB **S**oftware **L**ibrary (FSL; v.5.0), CSF partial volume estimate was (a) thresholded at 99% (eliminating voxels with <99% of their volume comprising CSF), (b) converted to a binary CSF mask, which was then (c) subtracted from the mask of a whole‐brain outline in order to generate a mask of total brain tissue that excluded the ventricular system. Brain volume masks underwent additional (d) minor manual editing using ITK‐SNAP (v.3.6.0; www.itksnap.org),^29^ to draw‐in any missing brain tissue. T1‐weighted data were used previously to report qualitative and quantitative findings regarding total brain and corpus callosum volumes.^19^

#### T2‐weighted total brain segmentation

**Figure 3:**
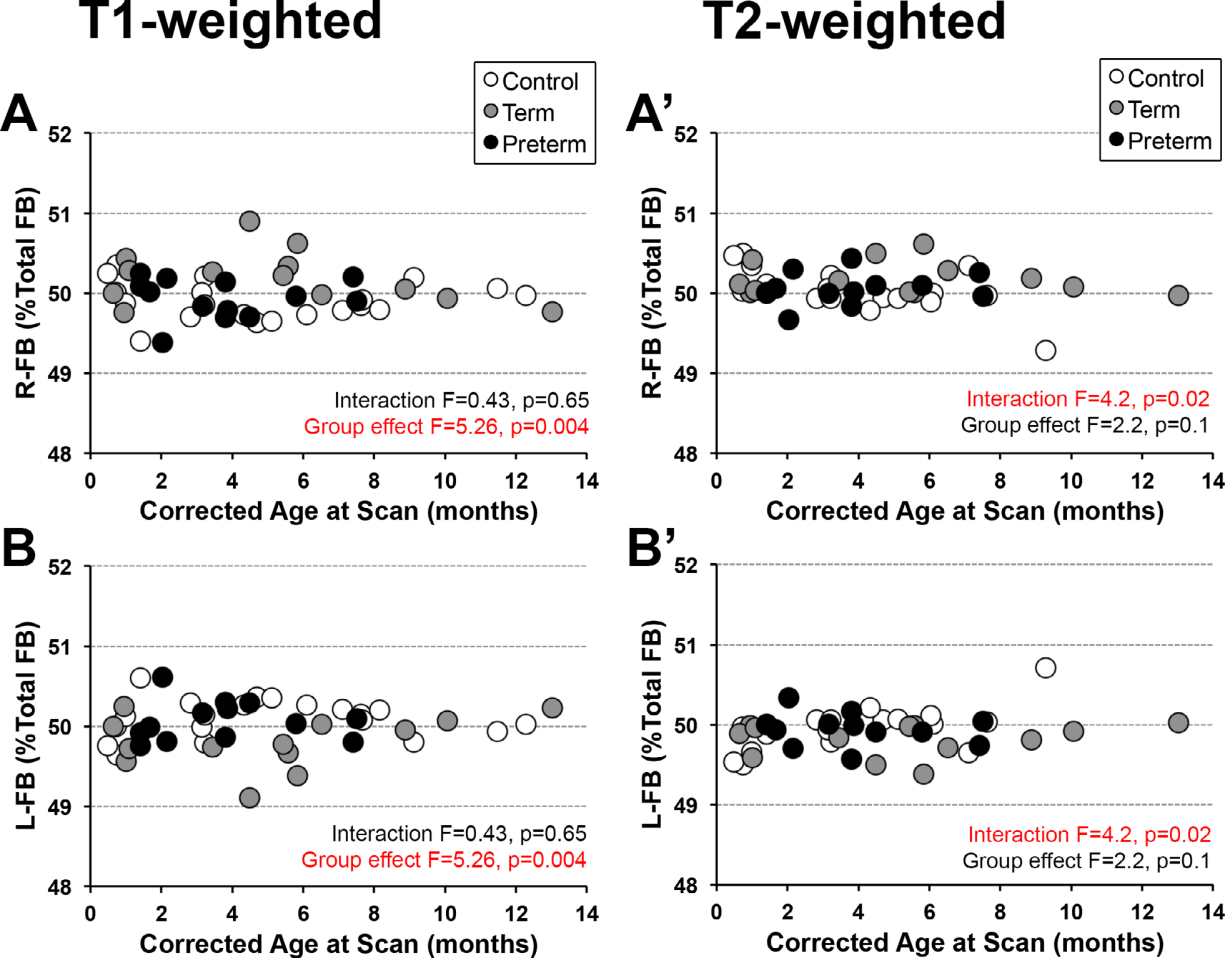
Normalized Hemispheric Volumes as a Percent of Total Forebrain Volume. Graphs show individual normalized hemispheric volumes as % total forebrain (FB) for the 3 groups: (1) term‐born controls (open circles), (2) term‐born patients (gray circles), and (3) premature patients (black circles) for T1‐ (A and B) and T2‐weighted (A’ and B’) structural MRI analyses. Lack of an age effect for all normalized variables suggests that hemispheres volumes change in proportion to the forebrain as a whole, irrespective of T1‐ vs. T2‐weighted modality (see Results section for statistical details). Despite this, one can qualitatively observe a tendency for *left‐greater‐than‐right asymmetry* for term‐born controls (open circles) in T1‐weighted analysis. Furthermore, T1‐weighted analysis shows group differences in normalized hemispheric volumes between groups (*F*(2,42) = 5.3, *p* = 0.009) with premature patients being different to controls (*p* = 0.004) and term‐born patients (*p* = 0.015), with no difference between term‐born patients and controls (*p* = 0.82). In contrast, T2‐weighted analysis (A’ and B’) does not show any group differences in normalized hemispheric volumes (*F*(2,39) = 2.2., *p* = 0.1), but shows significant interaction between age at scan and group status (*F*(2,37) = 4.2, *p* = 0.02), implicating altered hemispheric proportion with age between groups with time. Care should be put into interpretation of normalized results because of (i) the small range values (<2% difference between left and right hemispheres), (ii) less data points for older infants (e.g. premature infants older than 8 months), and (ii) the fact that term‐born patients (gray circles) show greater variability. *Abbreviations:* FB, forebrain; L, left; R, right.

**Figure 4:**
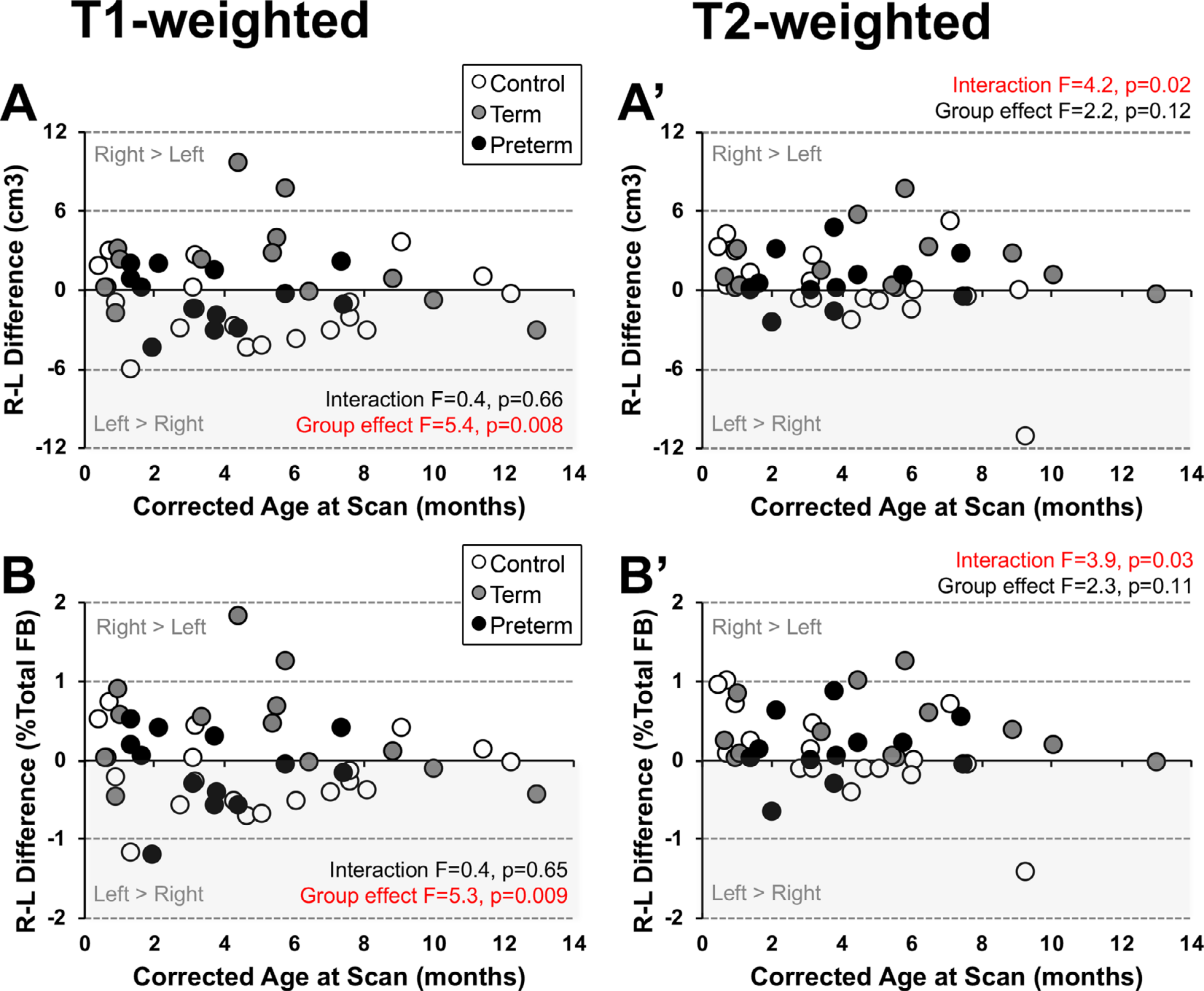
Predominant Hemispheric Asymmetry. Corresponding graphs show individual right minus left (R‐L) hemisphere absolute and normalized volume difference for the 3 groups: (1) term‐born controls (open circles), (2) term‐born patients (gray circles), and (3) premature patients (black circles) for T1‐ (A and B) and T2‐weighted (A’ and B’) MRI analyses. We report no significant trend for hemispheric asymmetry with age over the first year of life as analyzed by either structural MRI modalities (see Results section for statistical details). Note a qualitative tendency for *left‐greater‐than‐right* asymmetry for the term‐born controls (open circles in Panel A) in T1‐weighted analysis (more negative data points in R‐L difference). Furthermore, T1‐weighted analysis shows group differences in absolute and normalized hemispheric asymmetry (*F*(2,42) = 5.4, *p* = 0.008, absolute; *F*(2,42) = 5.3, *p* = 0.009 normalized) for premature patients compared to term‐born patients (*p* < 0.021) and controls (*p* < 0.004), with no difference between term‐born patients and controls (*p* > 0.60). In contrast, T2‐weighted analysis does not show any group differences in absolute (Panel A’) or normalized (Panel B’) hemispheric volume (absolute volume difference *F*(2,39) = 2.3., *p* = 0.11; normalized volume difference *F*(2,39) = 2.2, *p* = 0.12), but shows significant interaction between age at scan and group status (absolute volume difference *F*(2,37) = 3.9, *p* = 0.03; normalized volume difference *F*(2,37) = 4.2, *p* = 0.02) implicating altered trajectories for hemispheric dominance between groups with time. *Abbreviations:* FB, forebrain; L, left; R, right.

Methodology for preprocessing and segmentation of T2‐weighted data utilized Morphologically Adaptive Neonatal Tissue Segmentation (MANTiS) toolbox,^31^ as previously described in detail.^13^, ^17^ Preprocessing steps included: (i) Intracranial space segmentation: T2‐weighted images were skull stripped using the unvalidated “Simple Watershed Scalping” module in the MANTiS toolbox followed by manual editing in FSLview; (ii) Bias field correction using FMRIB’s Automated Segmentation Tool (FAST)^30^; (iii) Setting image origin using “Origin to the Center of Mass” module in the MANTiS toolbox. Preprocessed intracranial images underwent the MANTiS segmentation pipeline,^31^ which produced probabilistic tissue segmentations. Analysis of CSF was needed for calculation of total brain mask as the difference between intracranial space and CSF volumes (*Fig. 4 in* Ref. [13]). T2‐weighted data regarding total brain and CSF volumes were reported previously.^13^ As part of the gross regional brain segmentation using T2‐weighted images, forebrain volumes were previously calculated as: total brain volume − (cerebellum + brainstem) volumes (*Fig. 3 in* Ref. [17]).

#### Forebrain and hemispheric segmentation

Total forebrain masks for T1‐weighted images were created by manually erasing the cerebellum and brainstem from total brain mask using ITK‐SNAP. Total forebrain masks for T2‐weighted images were created by subtracting respective cerebellum and brainstem masks from the total brain mask using *fslmaths* with ‐suboperation (*see 3D illustration of forebrain mask in Fig. 1B in* Ref. [17]). Subsequently, left hemispheres were manually erased from total forebrain masks to create right hemisphere masks (Fig. 1). Volumes of total forebrain and right hemisphere segmentations were obtained using ITK‐SNAP volume estimation tool, and volume of the left hemisphere was calculated as the difference between total forebrain and the right hemisphere masks. Volumes are presented as *absolute* (cm^3^) and *normalized* (% forebrain volume). For the best visualization of expected *left‐greater‐than‐right* hemispheric asymmetry of infancy,^1^, ^3^, ^4^ data were also presented as absolute (cm^3^; Fig. 2) and normalized (% total forebrain; Fig. 3) volumes, as well as absolute and normalized volume *difference* between right and left hemispheres as previously validated by Shaw et al. 2009^32^ (Fig. 4). Specifically, % difference was calculated as [(Absolute difference between right and left hemisphere/average) × 100].

### Statistical analysis

As this was an extension of our pilot study and no prior information was available regarding the brain volumes in the selected cohort of infants following LGEA repair, a convenience sample size of 13 patients/group was chosen, based on the anticipated number of eligible infants at our institution and an estimated 50% enrollment rate. Statistical analyses were performed using the Statistical Package for the Social Sciences (SPSS, v.23.0; IBM Corporation, Armonk, NY). Normal distribution of all continuous variables was confirmed using the Shapiro‐Wilk test. To account for the potential confounding variable of having subjects scanned at various ages throughout the first year of life, comparison of volumes between the three groups was assessed using a general linear model (GLM) univariate analysis with corrected age at scan as a covariate and Bonferroni adjusted p values. The interaction (viz. test of parallelism) was reported when significant. Statistical significance was assessed at the α < 0.05.

## Results

Both T1‐ and T2‐weighted MRI data allowed for quantitative analysis of hemispheric volumes in term‐born and premature patients (n = 13/group), and term‐born controls (n = 20 for T1‐ and n = 17 for T2‐weighted analyses; Table 1). Total body weight and volumetric analysis of intracranial space, total brain, and cerebrospinal fluid (including its relative distribution in the extra‐axial space and ventricular system) of the current cohort were presented previously.^13^, ^17^

### Absolute volumes of the forebrain and its hemispheres

#### Size in infancy

As graphically illustrated in Figure 2, absolute volumes (cm^3^) increased with advancing age irrespective of the group status in both T1‐ and T2‐weighted analyses (Fig. 2). Specifically, absolute total *forebrain* volumes increased with age as per T1‐weighted (*F*(1,42) = 207.0, *p* < 0.001; Fig. 2A) and T2‐weighted analysis (*F*(1,39) = 169.7, *p* < 0.001; Fig. 2A'). Similarly, absolute volumes of right hemisphere (*F*(1,42) = 203.3, *p* < 0.001 for T1‐weighted; *F*(1,39) = 166.2, *p* < 0.001 for T2‐weighted) and left hemisphere (*F*(1,42) = 209.6, *p* < 0.001 for T1‐weighted; *F*(1,39) = 172.1, *p* < 0.001 for T2‐weighted) increased with age for all groups.

#### Interaction between age and group status

We also performed the analysis of group slope parallelism that implicates relative growth trajectories. T1‐weighted analysis failed to detect any significant interaction between age at scan and group status, indicating similar growth trajectory between the groups for total forebrain (*F*(2,40) = 1.6, *p* = 0.22; Fig. 2A), right hemisphere (*F*(2,40) = 1.6, *p* = 0.21; Fig. 2B), and left hemisphere (*F*(2,40) = 1.5, *p* = 0.23; Fig. 2C) volumes. In contrast, T2‐weighted analysis showed a significant interaction between age at scan and group status for absolute total forebrain (*F*(2,37) = 5.6, *p* = 0.008; Fig. 2A'), as well as right (*F*(2,37) = 4.9, *p* = 0.013; Fig. 2B') and left (*F*(2,37) = 6.3, *p* = 0.004; Fig. 2C') hemispheric volumes, suggesting altered growth trajectories between groups with advancing age.

#### Group differences

We report significant differences in absolute volumes for total forebrain and its hemispheres with smaller volumes in both term‐born and premature patients in comparison to controls (both *p* < 0.001), with no difference between patient groups (*p* > 0.7) in both types of analysis (viz. T1‐ and T2‐weighted data; Fig. 2). Of note, total forebrain data matches previously reported T2‐weighted volumes,^17^ with the addition of two control scans.

### Normalized volumes of hemispheres

#### Size in infancy

Neither right (*F*(1,42) = 0.6, *p* = 0.46 for T1‐weighted; *F*(1,39) = 3.6, *p* = 0.06 for T2‐weighted) nor left (*F*(1,42) = 0.6, *p* = 0.46 for T1‐weighted; *F*(1,39) = 3.6, *p* = 0.064 for T2‐weighted) hemisphere’s normalized volumes (% total forebrain) showed a significant change with advancing age for either T1‐ or T2‐weighted analysis. Such data suggest hemispheric volume to remain in proportion to the whole brain irrespective of the age, as graphically represented in Figure 3.

#### Interaction between age and group status

In analyzing group slope parallelism of the normalized volumes, T1‐weighted data analysis failed to detect any interaction between age and groups, indicating that reciprocal hemispheric proportion is maintained with age (*F*(2,40) = 0.4, *p* = 0.65; Fig. 3A and B). In contrast, T2‐weighted data (with smaller number of controls, including fewer controls at older ages) showed a significant interaction between age at scan and group status for reciprocal normalized hemispheric volumes (*F*(2,37) = 4.2, *p* = 0.02; Fig. 3A' and B') implicating (mal)adaptations in hemispheric proportion with age.

#### Group differences

Significant group differences for normalized volumes varied with respect to the type of analysis: T1‐ vs. T2‐weighted data analysis (Fig. 3). For T1‐weighted analysis (with larger power for controls; n = 20), we report significant differences in normalized hemispheric volumes (*F*(2,42) = 5.3, *p* = 0.009) with larger right hemispheres—and reciprocally smaller left hemispheres—in preterm patients in comparison to term‐born patients (*p* = 0.015) and controls (*p* = 0.004), and without differences between term‐born patients and controls (*p* = 0.82). Although term‐born patients (gray circle marker in Fig. 3) show a trend toward hemispheric volume reversal (right‐greater‐than‐left hemisphere), no significant difference was noted probably due to larger data variability. Interestingly, T2‐weighted analysis (with smaller number of controls; *n* = 17) did not show group differences with respect to normalized hemispheric volumes of preterm patients (*F*(2,39) = 2.2, *p* = 0.12) in comparison to term‐born patients (*p* = 0.086) and controls (*p* = 0.06). Normalized data results should be interpreted with caution, given the small % range difference and lack of data points for premature infants older than 8 months.

### Hemispheric asymmetry of infancy

To further characterize forebrain hemispheric predominance in this pilot study cohort of critically ill infants following LGEA repair with Foker process, we also show (i) absolute hemispheric difference (right minus left hemispheric volume difference; cm^3^)^32^ and (ii) normalized hemispheric difference^33^ (% total forebrain) for individual subjects (Fig. 4).

#### Size with age

The range of right minus left absolute hemispheric volume difference is small across the 1st year of life (<8 cm^3^; Fig. 4A and A'). There is a trend of *left‐greater‐than‐right* hemisphere in controls, and tendency for reversal in older controls (white circle markers in Fig. 4A and B for T1‐weighted analysis). However, we report no significance regarding hemispheric asymmetry with age for either T1‐weighted (absolute difference *F*(1,42) = 0.5, *p* = 0.49; normalized difference *F*(1,42) = 0.6, *p* = 0.46; Fig. 4A and B) or T2‐weighted analysis (absolute difference *F*(1,39) = 2.4, *p* = 0.13; normalized difference *F*(1,39) = 3.6, *p* = 0.06; Fig. 4A' and B').

#### Interaction between age and group status

Similar to results described above, T1‐weighted analysis did not detect any interaction between age and groups, while, T2‐weighted analysis findings suggest altered trajectories of *left‐greater‐than‐right* asymmetry in infancy among study groups (Fig. 4A' and B'). Note that individual normalized right‐left volume differences were in the 0‐2% range for both analyses (Fig. 4B and B').

#### Group differences

Analysis of group differences for absolute and normalized right minus left volume differences varied with respect to the type of analysis. T1‐weighted hemispheric volume difference for absolute (*F*(2,42) = 5.4, *p* = 0.008; Fig. 4A) and normalized (*F*(2,42) = 5.3, *p* = 0.009; Fig. 4B) values were significantly different in premature patients in comparison to term‐born patients (absolute difference *p* = 0.021; normalized difference *p* = 0.015) and controls (absolute difference *p* = 0.003; normalized difference *p* = 0.004), and exhibited a reversal of the expected left‐greater‐than‐right trend. While term‐born and premature patients show more similar trends of hemispheric asymmetry reversal (Fig. 4A and B), no significant group differences were noted between term‐born patient groups and controls (absolute difference *p* = 0.60; normalized difference *p* = 0.82). T2‐weighted hemispheric volume difference showed no group differences (Fig. 4A' and B').

## Discussion

Building on our previous findings from this same cohort which demonstrated smaller total forebrain volumes,^17^ our current pilot study shows proportionally smaller forebrain hemisphere volumes (cm^3^) in term‐born and preterm patients *after* complex thoracic non‐cardiac perioperative critical care involving prolonged sedation in comparison to term‐born controls. Despite small % difference in normalized hemispheric volumes, both T1‐ and T2‐weighted analyses implicate alterations in hemispheric asymmetry for infant patients following LGEA repair. Normalized hemispheric volumes (% total forebrain) and calculated right minus left hemispheric volume difference to suggest premature patients are more likely to deviate from the expected *left‐greater‐than‐right* hemispheric asymmetry of infancy.

### Absolute and normalized volumes of forebrain hemispheres

Irrespective of brain MRI modality used (T1‐ vs. T2‐weighted), absolute volumes of right and left forebrain hemispheres were proportionally smaller in both term‐born and premature patients after Foker process for LGEA repair in comparison to term‐born controls (Figure 2). This finding is in line with our previously published results of smaller total brain and forebrain volumes as demonstrated by T1‐^19^ and T2‐weighted analyses.^13^, ^17^ Normalized hemispheric volume group differences were only detected by T1‐weighted analysis (Figs. 3 and 4), in which preterm infants demonstrated right‐greater‐than‐left hemisphere volumes in comparison to term‐born patients or controls. While normalized group differences were very subtle (a right hemispheric predominance of roughly 2% of forebrain volume), they represent a deviation from the expected (i.e. typical) pattern of hemispheric brain asymmetry, where normal infants typically display a *left‐greater‐than‐right* predominance of roughly 4% of forebrain volume.^1^ Previous studies have already indicated prematurity may be a risk factor for abnormal hemispheric development: regarding total hemispheric volume,^9^ as well as in asymmetry localized to smaller areas such as the hippocampus.^34^ In contrast to our previous reports of clinically significant incidental brain MRI findings and smaller brain volumes in term‐born patients of the same cohort^13^, ^18^ we report no significant change in left‐greater‐than‐right hemispheric asymmetry following LGEA repair in term‐born patients. This discrepancy could be explained by small statistical power in the context of larger data variability for term‐born patients, who displayed trends similar to premature infants (Figs. 3 and 4). With the exclusion of extreme prematurity (see Methods), we failed to show a significant association between gestational age and brain asymmetry for either premature or term‐born patient groups (Kagan and Bajic, preliminary data) calling for additional research to evaluate prematurity as a risk factor for altered expected *left‐greater‐than‐right* hemispheric asymmetry in infancy. Future longitudinal studies with larger power should also include the control group of premature infants that did not undergo LGEA repair to help elucidate if the reported alteration in hemispheric asymmetry in infants born with LGEA are due to prematurity, complexity of perioperative care, or a combination of both. Additionally, the exact mechanisms behind these patterns of altered asymmetry are unclear. Previous studies have attributed normal neonatal asymmetry patterns to prenatal genetic programs.^35^ Therefore, it is possible that genetic predispositions along with postnatal environmental stress may affect early asymmetrical development in patients undergoing complex perioperative care for LGEA repair.

### Neurobehavioral sequelae of (mal)adaptations in forebrain asymmetry

Neurobehavioral implications of (a)typical forebrain hemispheric asymmetry in the studied cohort are not known. To date, the majority of the literature has explored hemispheric asymmetry patterns in healthy infants,^1^, ^2^, ^36^ important for establishing typical developmental patterns of brain asymmetry. Only one recent study of 16 extremely premature infants reported that reduced brain asymmetry observed at 40 weeks GA is potentially related to autism‐spectrum disorders upon long‐term follow up at 6.5 years of age.^9^ Other studies have linked abnormalities in hemispheric asymmetry to autism, developmental language disorder, and attention deficit hyperactivity disorder later in life (5‐21 years of age).^7^, ^37^ Disruption of asymmetry has also been implicated in the pathogenesis of other neurodevelopmental disorders, such as schizophrenia^38^, ^39^, ^40^ and developmental stuttering,^41^ while reduction of frontal lobe asymmetry in the context of smaller total brain and cerebral volumes was reported for children with pediatric post‐traumatic stress disorder.^42^

Few studies have also linked neonatal brain abnormalities and poor brain growth in extremely premature infants to autism spectrum disorder.^43^, ^44^ Consistent with previously published findings in our pilot cohort^13^, ^17^, ^19^ a recent 2017 study by Stolwijk et al.^14^ reported a high incidence of brain injury (viz. non‐parenchymal abnormalities, including intraventricular and subdural hemorrhages) in patients following neonatal surgery for major non‐cardiac congenital anomalies including esophageal atresia,^14^ as well as neurodevelopmental delay at 2 years of age,^15^ suggesting long‐term adverse neurodevelopmental sequelae in the setting of critical illness and non‐cardiac surgery in infancy. To our knowledge, no studies as of yet have evaluated the neurodevelopmental outcomes in either term‐born or premature infants following complex perioperative critical care with Foker process for LGEA repair. As such, our findings emphasize the necessity of long‐term follow up in the presented cohort of infants undergoing complex perioperative critical care involving prolonged sedation to assess whether (i) reported reversal of expected asymmetry in preterm infants persists into childhood, and (ii) to characterize the neurodevelopmental implications of such findings.

### Study limitations

#### Methodological considerations

We found slight discrepancies in the results between our two modes of analysis. Although both methodologies (T1‐ and T2‐weighted analysis) suggest altered *left‐greater‐than‐right* hemispheric dominance in our pilot cohort, we report significant group differences only in T1‐weighted analysis and significant interaction between age at scan and group status only in T2‐weighted analysis. There is contradictory evidence in the literature as to which modality is more appropriate for infant brain analysis,^45^, ^46^, ^47^ and decisions seem to be made on a case‐by‐case basis. Our T2‐weighted images, which offered higher in‐plane resolution and superior tissue contrasts, may be an important consideration for future studies assessing more detailed tissue segmentations that require delineation of grey and white matter boundaries. However, for the scope of this study, T1‐weighted images that offered better overall spatial resolution compared to T2‐weighted data were thought to provide more reliable information regarding gross hemispheric asymmetry patterns. Key differences between T1‐ and T2‐weighted analysis methodologies are summarized in Table 2.

#### Study power and sample size

It is possible that the lack of accordance between T1‐and T2‐weighted analyses in our study was due to fewer numbers of controls and, importantly, fewer controls at older ages (Fig. 4A and B). T1‐weighted analysis contained 20 controls with age ranges from 0.5‐12 months, whereas T2‐weighted analysis had 17 controls with age ranges from 0.5‐9.3 months (Table 1). Given the greater number of available scans with a wider age distribution, in addition to the methodological considerations mentioned above, T1‐weighted analysis was thought to provide a more complete and reliable assessment of hemispheric differences in the studied cohort. Since normalized asymmetry\x92differences were only in the 1% range for T2‐weighted analysis, should future investigations chose MRI data with poorer overall spatial resolution (2mm slices vs. 1mm for T1‐weighted analysis in our study), larger power should be recommended.

#### Other considerations

##### Additional controls

Future studies should strive to include a control group of LGEA patients that underwent alternative treatment, not including the Foker process, a group that received prolonged sedation with no surgery, or a group of premature infants that received no additional medical care.

##### Sex distribution

Uneven sex distribution in control groups (≥80% males) calls for future studies with more uniform sex distributions to account for possible sex‐differences in hemispheric asymmetry. Previous reports present inconsistent findings with regard to sexual dimorphisms in cerebral hemispheric asymmetry, with some studies reporting no sex differences in asymmetry,^48^, ^49^ and others reporting a significant sex‐effect.^50^

##### Timing of the MRI scans

MRI scans were not collected *prior* to Foker process treatment, so it is impossible to assess preexisting differences in left‐right asymmetry, or refute the possibility that detected alterations were due to prematurity alone and not critical illness and complex perioperative care.

## Conclusions

Our current pilot quantitative MRI study of hemispheric volumes suggests that premature patients might be at risk of altered expected *left‐greater‐than‐right* forebrain asymmetry in infancy. Future studies with larger power are needed to confirm findings in our pilot study data report. Neurobehavioral impact of described (mal)adaptations in brain asymmetry require future neurodevelopmental follow up in this unique infant population.

## Author Contributions

Authorship credit was based on substantial contributions to (1) the conception and manuscript design (CRLM and DB); (2) acquisition (CRLM, RWJ, and DB), analysis (MSK, CRLM, DZ, and DB), or interpretation of data (all authors); (3) drafting the article (MSK, CRLM, and DB) or critical revision for important intellectual content (all authors); (4) final approval of the version to be published (all authors); and (5) are accountable for all aspects of the work in ensuring that questions related to the accuracy or integrity of any part of the work are appropriately investigated and resolved (all authors).

## Conflict of Interest

None of the authors have any conflict of interest, including specific financial interests, relationships, or affiliations relevant to the manuscript.
